# Project Forward: A Population-Based Cohort Among Young Adult Survivors of Childhood Cancers

**DOI:** 10.1093/jncics/pkab068

**Published:** 2021-07-17

**Authors:** Joel Milam, David R Freyer, Kimberly A Miller, Jessica Tobin, Katherine Y Wojcik, Cynthia N Ramirez, Anamara Ritt-Olson, Stefanie M Thomas, Lourdes Baezconde-Garbanati, Michael Cousineau, Denise Modjeski, Sapna Gupta, Ann S Hamilton

**Affiliations:** 1 Department of Preventive Medicine, Keck School of Medicine, University of Southern California, Los Angeles, CA, USA; 2 Departments of Medicine and Epidemiology and Biostatistics, Chao Family Comprehensive Cancer Center, University of California, Irvine, CA, USA; 3 Children’s Hospital Los Angeles, Los Angeles, CA, USA; 4 USC Norris Comprehensive Cancer Center, Los Angeles, CA, USA; 5 Department of Dermatology, Keck School of Medicine, University of Southern California, Los Angeles, CA, USA; 6 VA Greater Los Angeles Health Care System, Los Angeles, CA, USA; 7 Public Health Sciences, Fred Hutchinson Cancer Center, Seattle, WA, USA; 8 Department of Epidemiology, University of Washington, WA, USA; 9 Department of Pediatric Hematology Oncology and Bone Marrow Transplantation, Cleveland Clinic Children’s Hospital, Cleveland, OH, USA

## Abstract

**Background:**

Childhood cancer survivors (CCS) face increased risk of morbidity and are recommended to receive lifelong cancer-related follow-up care. Identifying factors associated with follow-up care can inform efforts to support the long-term health of CCS.

**Methods:**

Eligible CCS (diagnosed between 1996 and 2010) identified through the Los Angeles County Cancer Surveillance Program responded to a self-report survey that assessed demographic, clinical, health-care engagement, and psychosocial risk and protective factors of recent (prior 2 years) cancer-related follow-up care. Weighted multivariable logistic regression was conducted to identify correlates of care. All statistical tests were 2-sided.

**Results:**

The overall response rate was 44.9%, with an analytical sample of n = 1106 (54.2% Hispanic; mean [SD] ages at survey, diagnosis, and years since diagnosis were 26.2 [4.9], 11.6 [5.4], and 14.5 [4.4] years, respectively). Fifty-seven percent reported a recent cancer-related visit, with lower rates reported among older survivors. Having insurance, more late effects, receipt of a written treatment summary, discussing long-term care needs with treating physician, knowledge of the need for long-term care, having a regular source of care, and higher health-care self-efficacy were statistically significantly associated with greater odds of recent follow-up care, whereas older age, Hispanic or Other ethnicity (vs non-Hispanic White), and years since diagnosis were associated with lower odds of recent care (all *Ps* < .05).

**Conclusions:**

Age and ethnic disparities are observed in receipt of follow-up care among young adult CCS. Potential intervention targets include comprehensive, ongoing patient education; provision of written treatment summaries; and culturally tailored support to ensure equitable access to and the utilization of care.

Improvements in childhood cancer treatment regimens have resulted in 5-year survival rates of more than 80% (1,2). Unfortunately, the majority of childhood cancer survivors (CCS) experience late adverse effects of cancer treatment, which often become clinically apparent years after treatment ends (3). Many of these late effects are severe or life-threatening and cause a range of symptomatic health problems, impaired function, and reduced quality of life (3-6). To facilitate prevention, detection, and management of late effects, the Children’s Oncology Group developed the Long-Term Follow-Up Guidelines for Survivors of Childhood, Adolescent and Young Adult Cancer, recommending that all CCS receive lifelong, risk-adapted surveillance and survivorship care (7).

Despite these recommendations, rates of health-care engagement among CCS decline with age and time since treatment, especially as CCS enter their 20s (5,8‐10). Because this attrition coincides with the rising cumulative incidence of late effects, it results in multiple missed opportunities for primary and secondary prevention (8). Studies among racial and ethnic minority CCS are also needed (11‐14). Disparities in health-care utilization among CCS have been observed by ethnicity, with higher proportions of non-Hispanic Whites (vs Hispanic) reporting receipt of cancer-related follow-up care, an association not explained by health insurance coverage (8). However, such disparities are not observed uniformly, suggesting variation in study samples, or individual- and/or system-level factors associated with health-care access (15‐17). Underlying drivers of age- and race- and ethnicity-related disparities in CCS follow-up care need continued investigation, particularly among ethnically diverse and more recently treated cohorts, as prior studies of CCS have primarily included non-Hispanic Whites and CCS diagnosed before 1999 and thus have been treated before numerous advances in treatment and survivorship care practices (eg, the broader use of survivorship care plans and survivorship clinics) and the impact of the Afforda ble Care Act (ACA) (18,19).

Prior research among CCS on access to and utilization of cancer-related follow-up care has focused predominantly on sociodemographic and clinical factors and less on organizational and psychosocial factors. For example, little is known about how many CCS have a regular source of care, the types of providers seen for cancer-related follow-up care, and patient knowledge of their health-care needs, as well as their confidence in navigating the health-care system (ie, health-care self-efficacy). Understanding these factors and their association with health-care utilization in early adulthood will clarify opportunities for intervention to prepare and support young adult CCS for managing their health care independently.

To address these gaps, we assessed the prevalence of clinical, demographic, psychosocial, and care-related factors, as well as their associations with receipt of cancer-related follow-up care in a diverse, population-based cohort of young adult CCS. We hypothesized that health insurance, greater knowledge of follow-up recommendations, younger age, non-Hispanic White (vs Hispanic) ethnicity, and higher health-care self-efficacy would be associated with greater use of cancer-related follow-up care.

## Methods

### Study Population

The Project Forward Cohort is a cancer registry–derived, population-based study of risk and protective factors of cancer-related follow-up care among young adult CCS. Data on all cases were obtained from the Los Angeles Cancer Surveillance Program, which is the cancer registry for Los Angeles County (part of the Surveillance, Epidemiology, and End Results program). Eligible participants included CCS who were diagnosed up to 19 years of age between 1996 and 2010 in Los Angeles County, California, with any cancer diagnosis (stage 2 or greater, except for brain and melanoma, which included stage 1 or greater) and who were at least 5 years postdiagnosis and aged 18-39 years when the study was launched in 2015.

### Procedure

**Figure 1.**
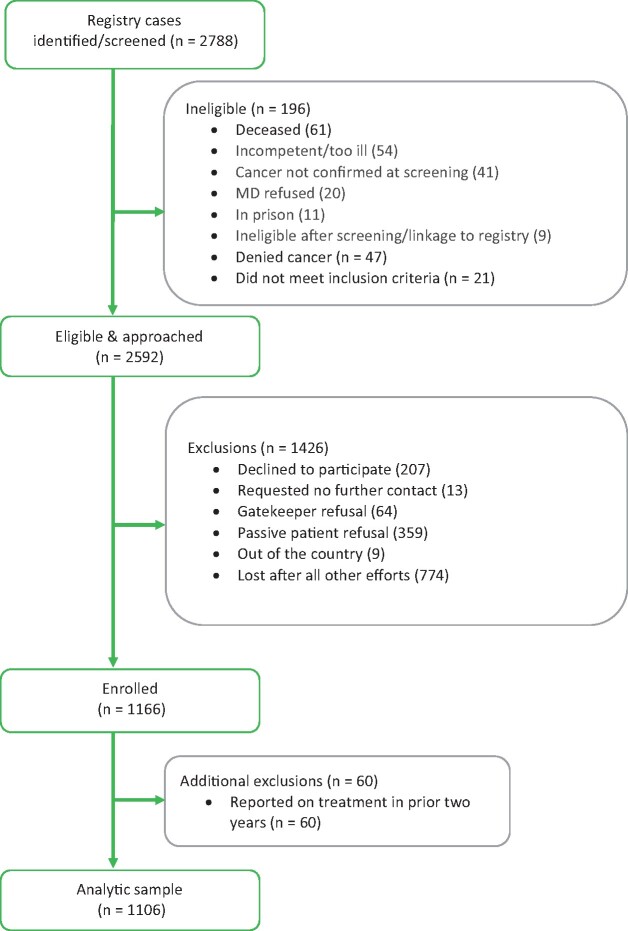
Project forward CONSORT diagram. MD = physician in registry record.

Recruitment methods were based on our pilot work (20) and included introductory postcards and self-report survey mailings in English and Spanish with the option to complete the survey online, over the phone, or in person in either language. Mailings also included a brochure describing the study and an informational brochure concerning the California Cancer Registry. Reminder mailings and calls occurred for those who did not respond. Although initial contact information (both recent address and address at diagnosis) is provided by the registry, we performed address tracing to improve accuracy of addresses and retraced potential participants who were difficult to contact (eg, in cases of post office returns) before being classified as lost after all efforts (see Figure 1). Participants received $20 cash and a lottery entry ($300). Participants who self-reported receiving cancer treatment less than 2 years prior to the study (n = 60) were excluded from analyses, with the exception of those with chronic myeloid leukemia due to the routine use of protracted maintenance therapy with tyrosine kinase inhibitors. Procedures were approved by the California State Committee for the Protection of Human Subjects, the institutional review board at the University of Southern California, and the California Cancer Registry.

### Measures

***Primary outcome.*** The primary outcome was receipt of cancer-related follow-up care in the prior 2 years (1 = yes, 0 = no). This was obtained via self-report and defined as any health-care visit where a provider completed an examination or tests to assess health problems from prior cancer or the cancer treatment they received, similar to an item used in the Childhood Cancer Survivor Study (5). Participants also indicated the type of health-care provider seen for this care (9).

***Demographic and clinical factors.*** Age at diagnosis, age at survey, cancer diagnosis (site and histology), diagnosing hospital, sex, race and ethnicity (non-Hispanic White, Hispanic, Asian, and other), and quintiles of neighborhood socioeconomic status (nSES) at diagnosis were obtained from the cancer registry. nSES is a census-based composite score (relative to California’s statewide distribution; 1 = lowest quintile nSES, 5 = highest quintile nSES), reflecting 7 indicators, including education index, percent persons above 200% poverty line, percent persons with a blue-collar job, percent persons employed, median rental, median value of owner-occupied housing unit, and median household income (21,22). Current health insurance (private, public, other, or uninsured) was self-reported.

As described in Intensity of Treatment Rating Scale 3.0, clinical and treatment information collected from medical charts is used to categorize cancer cases into 4 levels of treatment intensity (1 = least intensive [eg, surgery only]; 2 = moderately intensive [eg, chemotherapy or radiation]; 3 = very intensive [eg, 2 or more treatment modalities]; and 4 = most intensive [eg, relapse regimens]) (23). Because of the prohibitive cost of accessing medical charts for our large sample, we developed a novel method of calculating treatment intensity using cancer registry data as a proxy for chart data. Using our pilot study sample, for which treatment intensity had been previously determined using medical chart data, concordance between treatment intensity estimated by our method and treatment intensity estimated by the original chart-based method was assessed with Cohen Kappa statistic to validate this approach showing reasonable agreement between methods. A full description of the validation of this method of estimation of treatment intensity using cancer registry and self-reported data is available (24).

Self-reported late effects of cancer treatment included 11 items (eg, inability to have children, heart problems, difficulties with learning and memory, eyesight). Items were selected based on the most prevalent chronic conditions previously documented among CCS (3,25‐27). Summary scores were categorized as none, 1, or 2 or more late effects.

***Indicators of health-care engagement.*** These included having discussed future cancer-related health-care needs with any doctor, ever receiving a written cancer treatment summary, having a regular doctor for noncancer care, and ever sharing this written treatment summary with current doctors, which were separate, self-reported variables (1 = yes, 0 = no or not sure) (28).

Participants reported whether they believed they needed lifelong follow-up care (1 = yes, 0 = no or not sure).

***Psychosocial factors.*** Health-care self-efficacy (HCSE) was measured by 3 items related to perceived confidence in making appointments with physicians: obtaining cancer-related follow-up care; and discussing concerns with physicians, adapted from the Chronic Disease Self-Efficacy Scales from the Stanford Patient Education Research Center (29). Responses included “not at all confident” [0], “somewhat confident” [1], and “totally confident” [2] and were summed to create a total score that could range from 3 to 9 with higher scores representing greater HCSE (Cronbach alpha = 0.72).

Family influence was measured using a single item asking how often family has influenced the health-care decisions of the participants (1 = often or occasionally or 0 = never).

Depressive symptoms were assessed using the Center for Epidemiologic Studies Depression Scale (30). This scale includes 20 items about how often participants experienced symptoms in the past week, such as negative affect, sleep disruption, and feelings of hopelessness. Response options range from “rarely or none of the time” [0] to “most or all of the time” [3]. Scores were summed with a possible range of 0-60 (Cronbach alpha = 0.80) and dichotomized (1/0) at a score of 16 or greater to indicate likely depression.

### Statistical Analysis

Prevalence rates of the different components of survivorship care were examined both individually and cumulatively (to reflect receipt of multiple follow-up care recommendations) (31). This approach is similar to that used previously for identifying gaps in the implementation of recommended care for chronic diseases (eg, HIV, diabetes), using a prevalence-based “cascade of care” to highlight proportions receiving multiple dimensions of care (32,33).

Bivariate and multivariable logistic regression analyses were conducted to identify factors associated with receipt of cancer-related follow-up care. The multivariable model was weighted to account for survey response bias (correcting for differences in the distribution of sex, race and ethnicity, and nSES between survey responders and nonresponders) (34). Diagnosing hospital data were obtained from the cancer registry, and we incorporated clustered standard errors in all models to control for within-hospital correlations related to follow-up care.

Age at survey, sex, and race and ethnicity (as a proxy for unmeasured cultural and societal factors known to impact health-care access) were adjusted for in the multivariable model (35). The entry criteria for other variables to be retained in the multivariable model were a relationship with follow-up care in bivariate analyses (*P* ≤ .10) (36), which included years since diagnosis, nSES, health insurance, number of late effects, treatment intensity, receipt of a written cancer treatment summary, having a regular doctor for noncancer care, discussion of needed follow-up care with doctor, knowledge of the need for long-term follow-up care, HCSE, and family influence over health-care decisions. Depressive symptoms scores were dichotomized (0/1) at the clinical cut-point of 16, suggestive of depression. Health insurance was dichotomized to insured or uninsured because there was no statistically significant difference in follow-up care between public and private insurance statuses, and less than 2% of the sample reported “other” insurance. The variable, “shared a written treatment summary with current doctor,” was included in the cascade of care for descriptive purposes but was excluded from the final model because of its linear dependence on receipt of a written treatment summary. Listwise deletion was used to handle missing data (as indicated). Statistical significance was determined as a *P*-value less than .05 for 2-sided hypothesis tests. Data analyses were conducted in SAS statistical software (SAS Institute Inc, version 9.4, Cary, NC).

## Results

Of 2788 eligible cases, 196 were subsequently deemed ineligible (eg, too ill or incompetent, deceased) and 774 were lost (ie, no valid contact information; Figure 1). We recruited 1166 respondents. The response rate (denominator excludes confirmed ineligible) was 44.9%. Among those successfully contacted (eg, verified address and phone; n = 1764), the participation rate (denominator excludes confirmed ineligible and lost) was 64%: 39.3% (n = 434) completed the survey online, 1.2% (n = 13) over the phone, and the rest on paper (n = 659); 1.2% (n = 13) responded in Spanish. Responder analyses were performed using available demographic (at time of sample selection) and clinical variables from the registry data (Table 1). There were no differences between nonresponders (n = 1426) and responders (n = 1166) in age at diagnosis, years since diagnosis, age, cancer diagnosis, or stage of disease. However, those who responded were more likely to be female (vs male) and non-Hispanic White and have higher (vs lower) nSES. Our analyses excluded those who self-reported as on treatment within the prior 2 years (non–chronic myeloid leukemia, n = 60), and the final analytic sample size was 1106 (diagnosed across 68 sites).

**Table 1.** Differences between study responders and nonresponders on cancer registry variables (n = 2592 diagnosed in 1996-2010; Los Angeles County)

Characteristic	Nonresponder (n = 1426)^a^	Responder (n = 1166)^b^	Test statistic	
			χ^2^	*P* ^c^
Age at diagnosis, y			4.64	.20
0-4	187 (56.8)	142 (43.2)		
5-9	281 (56.4)	217 (43.6)		
10-14	361 (51.4)	342 (48.7)		
15-19	551 (55.5)	442 (44.5)		
Years since diagnosis (2015)			.0268	.99
5-9	358 (54.7)	296 (45.3)		
10-14	480 (55.1)	391 (44.9)		
15-22	588 (55.1)	479 (44.9)		
Sex			23.39	<.001
Male	834 (59.4)	571 (40.6)		
Female	592 (49.9)	595 (50.1)		
Age in 2015, y			3.32	.34
18-20	314 (56.7)	240 (43.3)		
21-25	538 (55.8)	427 (44.3)		
26-30	345 (52.0)	318 (48.0)		
31-39	229 (55.9)	181 (44.2)		
Race and ethnicity			29.68	<.001
Non-Hispanic White	309 (47.6)	340 (52.4)		
Hispanic	815 (57.3)	607 (42.7)		
Asian	106 (49.5)	108 (50.5)		
Other	196 (63.8)	111 (36.7)		
Cancer diagnosis			6.69	.24
Lymphoma	257 (51.3)	244 (48.7)		
Leukemia	479 (54.1)	407 (45.9)		
Brain and other nervous system	260 (58.7)	183 (41.3)		
Endocrine system	79 (53.74)	68 (46.26)		
Skin	60 (57.14)	45 (42.86)		
Other^d^	291 (57.1)	219 (42.9)		
Stage of disease (missing n = 2)			5.12	.16
Local	271 (57.7)	199 (42.3)		
Regional	398 (52.4)	361 (47.6)		
Distant	755 (55.5)	606 (44.5)		
Socioeconomic status			15.67	.004
Lowest	521 (59.1)	361 (40.9)		
Low	314 (55.1)	256 (44.9)		
Medium	225 (55.7)	179 (44.3)		
High	169 (47.5)	187 (52.5)		
Highest	197 (51.8)	183 (48.2)		

a Among those eligible and approached.

b Among those initially enrolled.

c Two-sided, χ^2^ tests.

d Oral cavity and pharynx, digestive system, respiratory system, soft tissue including heart, urinary system, eye and orbit, and miscellaneous.

**Figure 2.**
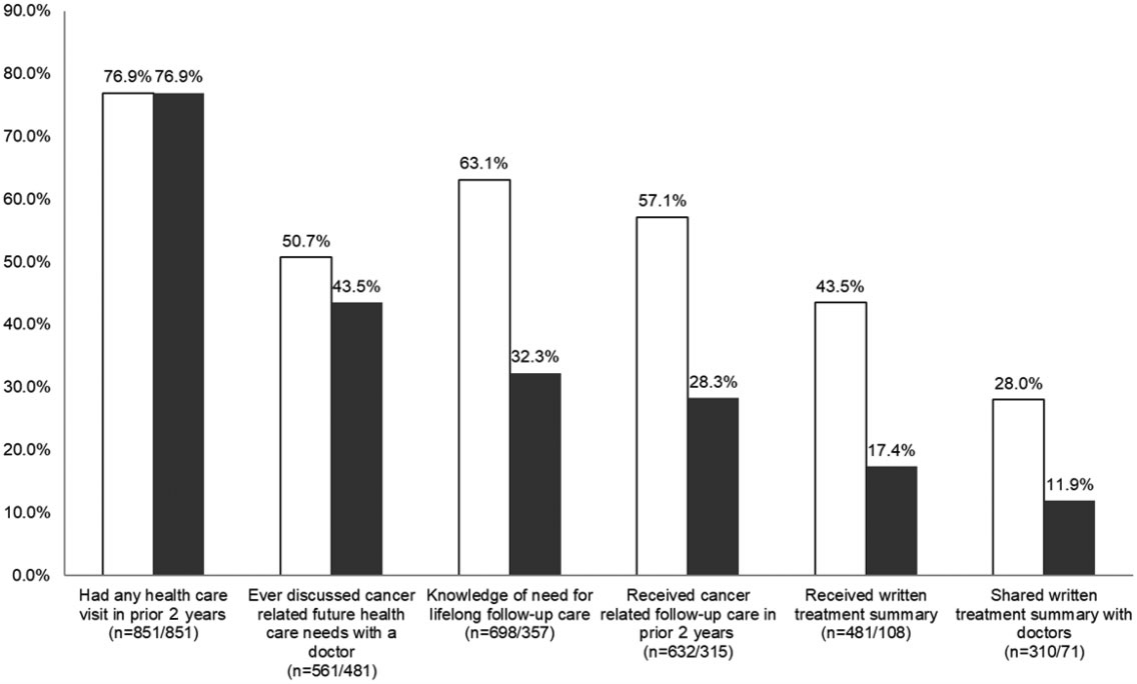
Cascade of recommended long-term follow-up care. Data reflect 1106 childhood cancer survivors in a population-based cohort of Los Angeles County (diagnosed in 1996-2010). Raw percentages (**white bars**) are mutually exclusive, and cascade percentages (**black bars**) are cumulative, from left to right (eg, the last column indicates that 11.9% of the sample answered yes for all categories). The sequence of care elements is based on clinician feedback and does not represent a prescriptive causal pathway.

Participants (54.2% Hispanic; 46.0% female) had a mean age of 11.6 (SD = 5.4) years at diagnosis and a mean age at survey completion of 26.2 (SD = 4.9) years (Table 2). At the time of survey, participants were an average of 14.5 (SD =4.4; range = 5-22) years from diagnosis. The most common cancer diagnoses included leukemia (36.1%), lymphoma (21.7%), and brain (15.2%). Of the participants, 57% reported a cancer-related follow-up care visit in the prior 2 years. The most common health-care providers for cancer-related follow-up care included adult oncologists (41.8%), pediatric oncologists (29.9%), and primary care physicians (15.5%) (not mutually exclusive). Rates of endorsement for key components of survivorship care, including discussing follow-up care, knowledge of the need for follow-up care, receiving a written treatment summary, and sharing that summary with doctors, individually ranged from 28.3% to 63.1% (Figure 2). Examining these indicators cumulatively, the survivorship cascade decreased at each step of care, resulting in 11.9% reporting yes to all measured follow-up care components (cumulative bars in Figure 2).

**Table 2.** Descriptive statistics of registry and self-report data from participants enrolled in the Project Forward Cohort (n = 1106)

Variable	No. (Weighted %)
Cancer registry data	
Age at diagnosis, y	
Mean (SD) [range]	11.60 (5.37) [0-19]
0-4	155 (14.3)
5-9	214 (19.5)
10-14	329 (29.8)
15-19	408 (36.5)
Years since diagnosis, y	
Mean (SD) [range]	14.54 (4.37) [5-22]
5-9	174 (15.7)
10-14	354 (31.7)
15-22	578 (52.6)
Sex	
Male	544 (54.0)
Female	562 (46.0)
Age at survey completion, y	
Mean (SD) [Range]	26.15 (4.87) [18-41]
Age group at survey completion, y	
18-20	131 (11.7)
21-25	422 (38.7)
26-30	339 (30.3)
31-41	214 (19.3)
Race and ethnicity	
Non-Hispanic White	324 (27.4)
Hispanic	570 (54.2)
Asian	107 (9.2)
Other	105 (9.2)^a^
Cancer diagnosis	
Leukemia	392 (36.1)
Lymphoma	240 (21.7)
Brain and other nervous system	169 (15.2)
Endocrine system	60 (5.1)
Bones and joints	56 (5.0)
Skin	41 (3.5)
Genital system	56 (5.0)
Other	92 (8.2)^b^
Treatment intensity^c^	
1 (least intensive)	69 (6.0)
2 (moderately intensive)	344 (30.9)
3 (very intensive)	544 (49.9)
4 (most intensive)	149 (13.3)
Socioeconomic status at diagnosis	
Lowest	344 (34.8)
Low	238 (21.2)
Medium	167 (14.6)
High	180 (14.6)
Highest	177 (14.8)
Self-report data^d^	
Health insurance (missing n = 35)	
Private	631 (57.2)
Public	321 (31.1)
Other/Unknown	17 (1.8)
None	102 (10.0)
Health-care self-efficacy (missing n = 20)^e^	
Mean (SD) [range]	4.83 (1.3) [0-6]
High levels of depressive symptoms (missing n = 93)^f^	353 (35.0)
Family influence health-care decisions (yes; missing n = 17)	935 (85.7)
Has doctor for regular (noncancer) health checkups (missing n = 19)	783 (71.4)
Had any health-care visit in prior 2 years (missing n = 0)	851 (76.4)
Discussed cancer-related follow-up care needs with a doctor (yes, in the last 2 years; missing n = 20)	561 (51.1)
Knowledge of need of lifelong follow-up care (missing n = 16)	698 (63.7)
Received cancer-related follow-up care (missing n = 19)	632 (57.7)
Received written cancer treatment summary (missing n = 20)	481 (43.9)
Shared written treatment summary with other doctors (missing n = 1)	310 (28.1)

a Including 53 Black, 39 Middle Eastern, 1 non-Hispanic, American-Indian, and 12 other/unknown.

b Oral cavity and pharynx, digestive system, respiratory system, soft tissue including heart, urinary system, eye and orbit, and miscellaneous.

c Intensity of Treatment Rating (based on both registry and self-report data, see Methods).

d Based on self-report data (all missing less than 5%, except for depressive symptoms, which was 8% missing).

e Examined as a continuous variable.

f Center for Epidemiological Studies-Depression score of 16 or greater.

**Table 3.** Univariate and multivariable logistic regression models of receipt of cancer-related follow-up care (within prior 2 years) among childhood cancer survivors (diagnosed in 1996-2010; Los Angeles County)^a^

Characteristic	Bivariate analyses		Multivariable model	
	OR (95% CI)	*P*	Adjusted OR (95% CI)	*P*
Years since diagnosis	0.88 (0.85 to 0.90)	<.001	0.88 (0.84 to 0.92)	<.001
Age at survey completion, y				
18-20	1.00 (referent)	—	1.00 (referent)	—
21-25	1.69 (1.31 to 2.19)	<.001	0.65 (0.50 to 0.85)	.002
26-30	0.70 (0.54 to 0.92	.01	0.32 (0.22 to 0.48)	<.001
31-39	0.45 (0.33 to 0.61)	<.001	0.35 (0.24 to 0.50)	<.001
Female (vs Male)	1.39 (1.09 to 1.77)	.01	1.16 (0.86 to 1.58)	.34
Race and ethnicity (relative to non-Hispanic White)				
Non-Hispanic White	1.00 (referent)	—	1.00 (referent)	—
Hispanic	0.81 (0.59 to 1.11)	.18	0.69 (0.51 to 0.95)	.02
Asian	0.75 (0.43 to 1.29)	.29	0.83 (0.52 to 1.31)	.42
Other	0.89 (0.61 to 1.31)	.54	0.69 (0.48 to 0.99)	.04
Socioeconomic status (relative to lowest group)				
Lowest	1.00 (referent)	—	1.00 (referent)	—
Low	0.89 (0.66 to 1.19)	.42	0.92 (0.66 to 1.26)	.59
Medium	1.06 (0.75 to 1.49)	.74	1.12 (0.76 to 1.65)	.56
High	1.59 (1.13 to 2.24)	.01	1.01 (0.67 to 1.52)	.97
Highest	1.09 (0.78 to 1.52)	.60	0.93 (0.62 to 1.39)	.73
Health insurance (any vs bone)	3.05 (1.96 to 4.75)	<.001	2.06 (1.28 to 3.32)	.003
High levels of depressive symptoms	0.93 (0.71 to 1.21)	.57	(not included)	—
No. of late effects (relative to none)				
0	1.00 (referent)	—	1.00 (referent)	—
1	1.23 (0.89 to 1.69)	.21	1.41 (1.08 to 1.83)	.01
≥2	1.51 (1.10 to 2.07)	.01	1.54 (1.23 to 1.92)	<.001
Treatment intensity	1.28 (1.10 to 1.50)	.002	1.18 (0.92 to 1.52)	.20
Received written cancer treatment summary	2.72 (2.10 to 3.52)	<.001	1.47 (1.16 to 1.87)	.002
Has doctor for regular (noncancer) health checkups	2.03 (1.54 to 2.67)	<.001	1.47 (1.13 to 1.92)	.005
Discussed cancer-related follow-up care needs with a doctor (in the last 2 years)	3.28 (2.54 to 4.24)	<.001	1.95 (1.49 to 2.55)	<.001
Knowledge of need of lifelong follow-up care	3.53 (2.71 to 4.60)	<.001	3.57 (2.90 to 4.39)	<.001
Health-care self-efficacy	1.35 (1.23 to 1.48)	<.001	1.23 (1.09 to 1.39)	<.001
Family influence health-care decisions	1.36 (0.96 to 1.93)	.08	0.90 (0.59 to 1.38)	.63

a All logistic regression models adjust for clustering at diagnosing hospital. All variables are included, and mutually adjusted for, in the multivariable model except for depressive symptoms (which was not statistically significant in the bivariate analyses). *P* values are 2-sided. CI = confidence interval; OR = odds ratio; — indicates no *P* value.

In the adjusted multivariable model (Table 3), years since diagnosis, current age, Hispanic and Other ethnicity (vs non-Hispanic White), and age at survey were statistically significantly negatively associated with follow-up care (all *Ps* < .05). Health insurance, number of late effects, receipt of a written treatment summary, having a regular doctor for noncancer care, discussion of needed follow-up care with physician, knowledge of the need for long-term follow-up care, and HCSE were all statistically significantly positively associated with receipt of recent care (all *P*s< .05).

In exploratory models, we examined multivariable models stratified by Hispanic ethnicity (Table 4). These results were largely consistent between Hispanics and non-Hispanics, with the exception of nSES, which showed a stronger positive association with receipt of recent care among non-Hispanics.

**Table 4.** Multivariable logistic regression models of receipt of cancer-related follow-up care (within prior 2 years), stratified by ethnicity (Hispanic, non-Hispanic)^a^

Characteristic	Hispanic (n = 570)		Non-Hispanic (n = 536)	
	OR (95% CI)	*P*	Adjusted OR (95% CI)	*P*
Years since diagnosis	0.87 (0.82 to 0.93)	<.001	0.88 (0.84 to 0.93)	<.001
Age at survey completion, y				
18-20	1.00 (referent)	—	1.00 (referent)	—
21-25	0.63 (0.37 to 1.07)	.08	0.66 (0.43 to 1.01)	.06
26-30	0.32 (0.18 to 0.55)	<.001	0.31 (0.20 to 0.48)	<.001
31-39	0.30 (0.13 to 0.66)	.004	0.44 (0.20 to 1.00)	.049
Female (vs Male)	1.00 (0.66 to 1.51)	.99	1.28 (0.81 to 2.03)	.28
Socioeconomic status (relative to lowest)				
Lowest	1.00 (referent)	—	1.00 (referent)	—
Low	0.64 (0.45 to 0.91)	.01	3.09 (1.14 to 8.37)	.003
Medium	0.92 (0.43 to 2.00)	.84	2.82 (1.50 to 5.31)	.001
High	0.75 (0.42 to 1.36)	.34	2.47 (1.09 to 5.57)	.03
Highest	1.57 (0.75 to 3.28)	.23	2.00 (0.91 to 4.38)	.08
Health insurance (any vs none)	1.53 (1.00 to 2.35)	.049	2.98 (1.06 to 8.32)	.04
No. of late effects (relative to none)				
0	1.00 (referent)	—	1.00 (referent)	—
1	1.15 (0.78 to 1.69)	.47	2.17 (1.47 to 3.19)	<.001
≥2	1.67 (1.10 to 2.53)	.02	1.72 (1.02 to 2.92)	.04
Treatment intensity	1.12 (0.65 to 1.94)	.67	1.17 (0.98 to 1.41)	.09
Received written cancer treatment summary	1.37 (1.04 to 1.79)	.03	1.64 (1.16 to 2.33)	.007
Has doctor for regular (non-cancer) health checkups	1.70 (1.20 to 2.40)	.004	1.24 (0.78 to 1.97)	.36
Discussed cancer-related follow-up care needs with a doctor (in the last 2 years)	2.40 (1.82 to 3.16)	<.001	1.44 (0.93 to 2.23)	.10
Knowledge of need of lifelong follow up care	3.93 (2.78 to 5.56)	<.001	3.70 (2.41 to 5.68)	<.001
Health-care self-efficacy	1.25 (1.08 to 1.45)	.004	1.25 (1.08 to 1.53)	.04
Family influence health-care decisions	0.69 (0.39 to 1.21)	.19	1.24 (0.77 to 1.98)	.38

a All models adjust for clustering at diagnosing hospital. All variables are included, and mutually adjusted for, in each multivariable logistic regression model. *P* values are 2-sided. CI = confidence interval; OR = odds ratio; — indicates no *P* value.

## Discussion

Long-term survivorship care is critical for health maintenance among CCS, but determinants of engagement in care are complex and vary by numerous patient- and system-level factors. This study leveraged a sociodemographically diverse, population-based sample to examine novel correlates of cancer-related health-care engagement, including health-care organizational factors. We found each care component to have a statistically significant (all *Ps* < .01) independent association with follow-up care, suggesting that each represents a unique indicator of engagement. However, only 12% of the sample endorsed all components, indicating the critical need for improvement of the full spectrum of survivorship care. Because receiving (43.9%) and sharing (28.1%) a written treatment summary were the least endorsed elements, these represent components amenable to improvement through practical interventions to increase utilization of care (37,38).

In addition to equipping CCS themselves with a thorough understanding of follow-up recommendations, their future, nononcology physicians who may not be familiar with recommended survivorship guidelines must also be supported. In a study of primary care physicians who cared for CCS, 48% had never or almost never received a cancer treatment summary, two-thirds were not comfortable caring for CCS, and few correctly identified guideline-recommended surveillance for sentinel late effects such as cardiac dysfunction (39). Those providers reported having access to clinical surveillance guidelines and receiving patient-specific information would be most likely to improve their quality care for survivors (39). Therefore, care coordination and information sharing between oncology and primary care physicians are needed to support survivors. Specialized cancer survivor programs are unlikely to fully support the growing number of CCS, and indeed, more than 15.5% of our sample reported seeing a primary care physician for their follow-up care, underscoring the importance of equipping primary care providers to care for this unique population.

Our findings demonstrate that although roughly half the sample reported recent cancer-related follow-up care, rates differed by race and ethnicity, consistent with prior research (8,9,18,40). Among Hispanics, the odds of reporting recent follow-up care were 31% lower compared with non-Hispanic Whites. This disparity was not explained by nSES, health insurance, or treatment differences, so additional factors need assessment to inform efforts to improve equity in access to care. For example, failure to adequately account for cultural characteristics and beliefs around health and disease in the provision of care may partially drive ethnic disparities by posing a barrier to patients’ understanding of health-care providers’ instructions (41). Other factors that may underlie ethnic differences in access to care include conceptions about Western medicine, fatalism, or risk perception (41‐44). Investigation of sociocultural factors (eg, culturally based beliefs about disease, language, understanding of insurance, neighborhood resources) mediating disparities in follow-up care among CCS is underway to clarify subgroups at greater risk of disengagement from care and potential areas to target tailored support (14).

The observed decline in rates of follow-up care with age (and years since diagnosis) is consistent with prior research showing a notable drop in the period of emerging adulthood (primarily occurring between ages 18 and 25 years) (5,8,18,45). In our study, the odds of recent care among those aged 31-41 years were 65% lower compared with those ages 18-20 years. CCS in their early 20s are especially vulnerable to the effects of interrupted health insurance due to the typical losses of state Children’s Health Insurance Program coverage at age 21 years and of parent-based private insurance coverage at age 26 years. Although passage of the ACA in 2010 expanded health insurance access for young adults, 10% of our cohort was uninsured, and having insurance was associated with 106% greater likelihood of reporting recent follow-up care. Because follow-up care remains suboptimal despite the widespread implementation of the ACA, future work should examine discontinuity of coverage, high deductibles, and/or partial coverage for screening as barriers to follow-up care.

Declines in health-care engagement with age are likely explained, in part, by competing developmental tasks, as young adulthood is a time marked by major transitions and acquired responsibilities (45). The transition from the pediatric oncology setting to adult-focused care should ideally include interprovider communication, involvement of family to discuss the transition of responsibility, and patient education to support health-care independence (eg, information regarding prior treatment exposure, health risks, health insurance, finding a new provider) (46‐48). However, survivors often transition by default through simply aging out of pediatric care, which leads to severe attrition to follow-up and reactive medical care (49). Standardized transition assessments and patient navigation systems may enable more CCS to successfully transition to, and remain engaged in, adult survivor–focused care as they age with unique health needs (50).

HCSE, the perceived ability to manage one’s health, was a statistically significant (*P* < .001) independent facilitator of follow-up care. HCSE may promote and be promoted by engagement in the health-care system. For example, attendance at a survivorship clinic equips survivors with greater knowledge about their disease, health risks, and preventive behaviors, which may contribute to greater self-efficacy (51). In turn, greater HCSE supports survivors in seeking out follow-up care and maintaining long-term surveillance. Research among adult cancer survivors has shown that receiving a verbal explanation of follow-up care plans was statistically significantly associated with higher HCSE, and higher HCSE was associated with lower rates of hospitalization, possibly because of the improved ability to manage health preventively (52). Enhancing HCSE through comprehensive patient education can support lifelong health management among CCS.

Strengths of this study include the ethnically diverse, recently diagnosed, population-based sample with rich survey data. Our response rate was similar to other registry-based epidemiologic studies of cancer survivorship, despite the challenges of recruiting a younger, more geographically mobile population with a longer time since diagnosis (53,54). We were able to address response bias by weighting our analyses on demographic factors related to response (eg, sex). Although we were unable to evaluate nonregistry variables associated with likelihood of study participation (eg, current insurance status), as they were unavailable for survey nonresponders, we believe recruitment bias in this cohort is substantially lower than hospital-based studies where study participants generally have greater health-care access.

Additional limitations include the cross-sectional nature of the data, which inhibits causal inference. For example, the positive association between late effects and follow-up care may be due to CCS seeking care because of late effects and/or health-care providers effectively identifying late effects. Analyses were restricted to those diagnosed in 1 geographical region and may not be generalizable to other areas (eg, with different geographically related characteristics related to health-care access). Additionally, the cascade of care does not reflect a unidirectional, prescriptive causal pathway. Longitudinal data are needed to clarify causal pathways to better understand optimal points of intervention to maximize the long-term health of CCS. Finally, more in-depth assessments of perceived risk, risk-based surveillance, and care received (eg, chart abstract data validating self-report, receipt of guideline-concordant screening exams) can further contextualize CCS knowledge and their health-care utilization and are the focus of ongoing work.

Long-term follow-up care is essential to mitigate the heightened risk of morbidity among CCS. With growing numbers of cancer survivors, greater efforts are needed to increase health-care engagement as survivors age and to minimize ethnic disparities in access. Based on these results, pragmatic approaches for promoting preventive health management among CCS include patient and provider education, written treatment summaries, and standardized plans for transitioning CCS from the pediatric to adult care setting.

## Funding

This work was supported by the National Institute on Minority Health and Health Disparities of the National Institutes of Health (grant number 1R01MD007801) and the National Cancer Institute (grant numbers P30CA014089, T32CA009492). Jessica Tobin was also supported by the VA Office of Academic Affiliations through the Advanced Fellowship Program in Health Services Research and Development. The contents do not represent the views of the U.S. Department of Veterans Affairs or the United States Government.

## Notes

**Role of the funder:** The study funders played no role in the design of the study; the collection, analysis, and interpretation of the data; the writing of the manuscript; and the decision to submit the manuscript for publication.

**Disclosures:** The authors have no conflicts of interest to disclose.

**Authorcontributions:** Conceptualization- JM, AH, DF. Data curation- JT. Formal analysis- JM, CR, JT. Funding acquisition- JM. Investigation- JM, DM, CR, JT, KW, AH. Methodology- JM, AH, CR. Project administration- JM, AH, DM. Resources- SG. Software- JT. Supervision- JM, AH, LB, MC. Visualization- JM, CR. Writing- original draft- JM, JT, CR. Writing- review & editing- JM DF KM JT KW CR AR ST LB MC DM SG AH.

**Priorpresentations:** This research was previously presented in part at the North American Symposium on Late Complications after Childhood Cancer, Atlanta, GA, June 2019.

**Acknowledgements**: We thank the participants for their involvement with this study.

## Data Availability 

The data underlying this article cannot be shared publicly due to privacy restrictions of individuals that participated in the study. Aggregated, deidentified data may be shared on reasonable request to the corresponding author.
